# Supplementary material for: Synthesis and Antiproliferative Potential of Thiazole and 4-Thiazolidinone Containing Motifs as Dual Inhibitors of EGFR and BRAFV600E
Source: Molecules. 2023 Dec 5;28(24):7951. doi: 10.3390/molecules28247951 (PMC10745574; doi:10.3390/molecules28247951)
Supplement: Supplementary file 1 [file molecules-28-07951-s001.zip › molecules-2685349-supplementary.pdf]

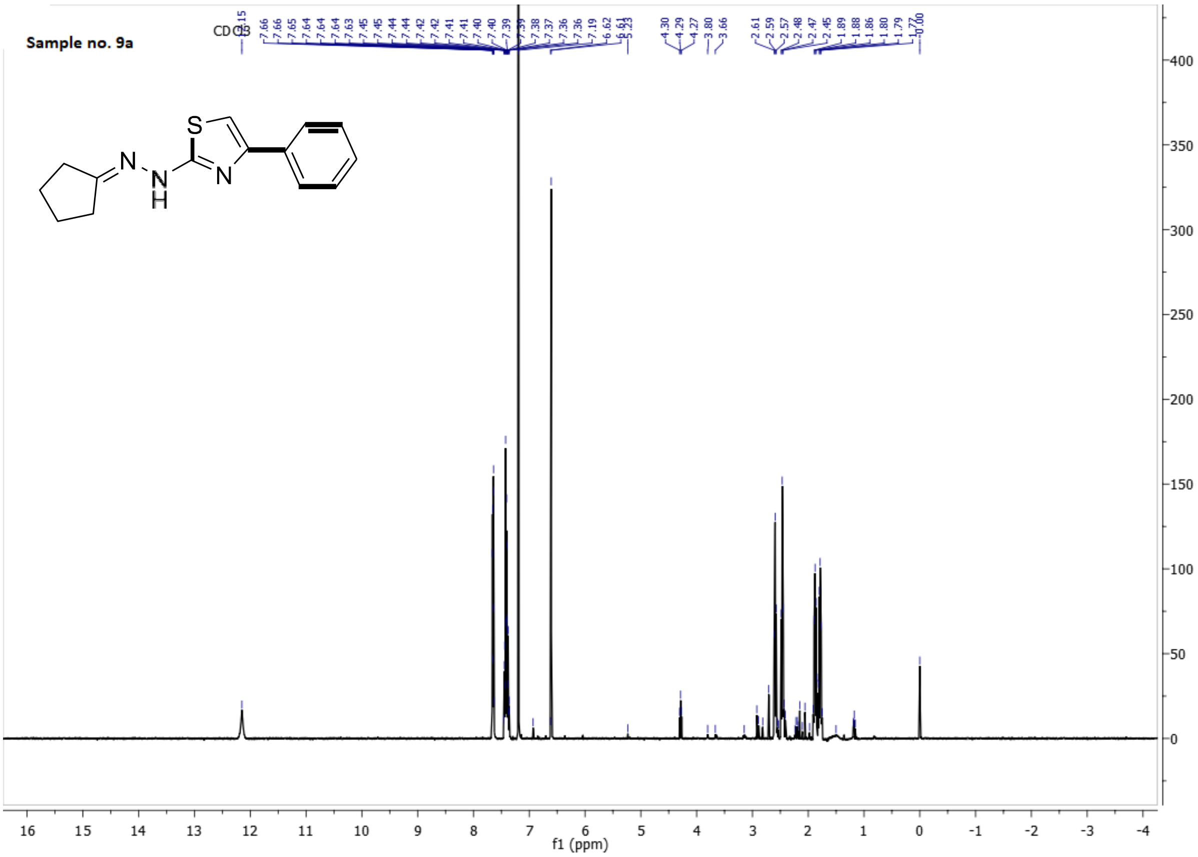

S1 ^{1}H NMR of 9a

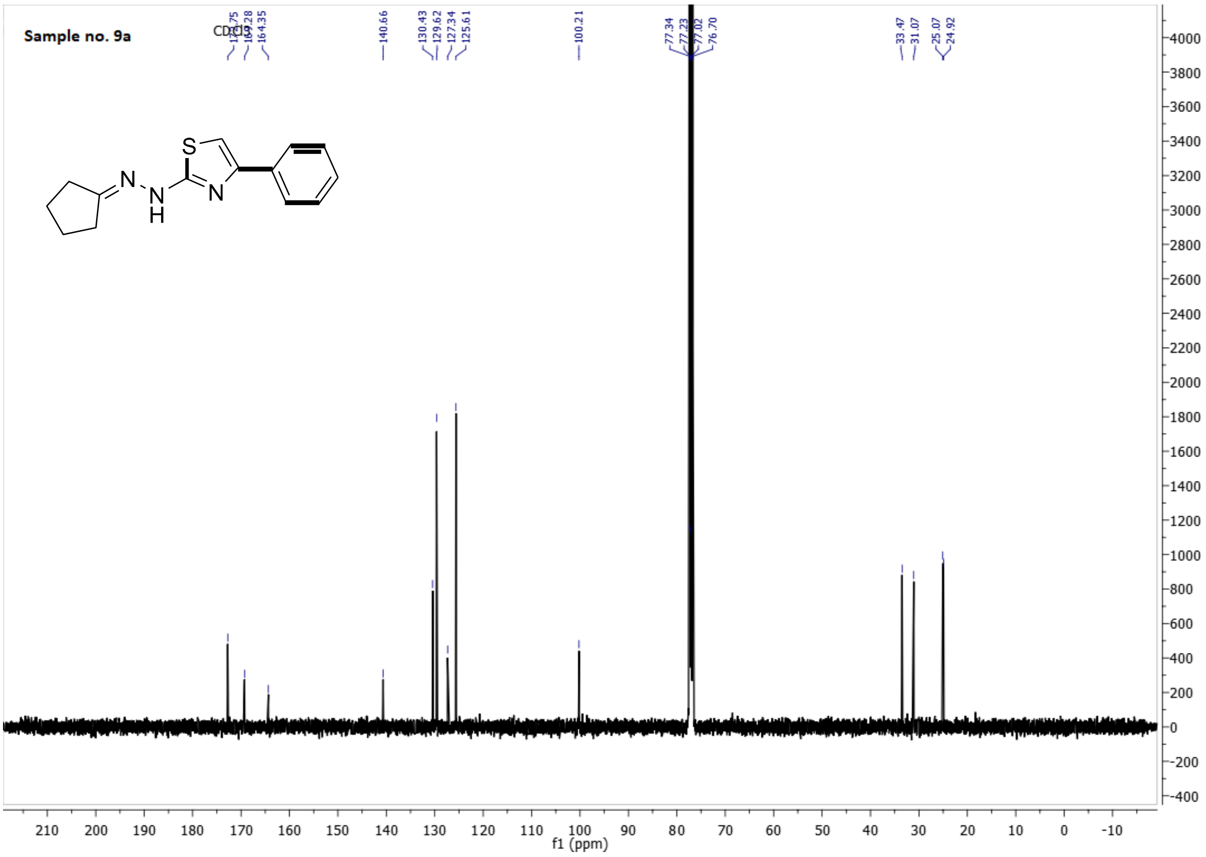

S2 ^{13}C NMR of 9a

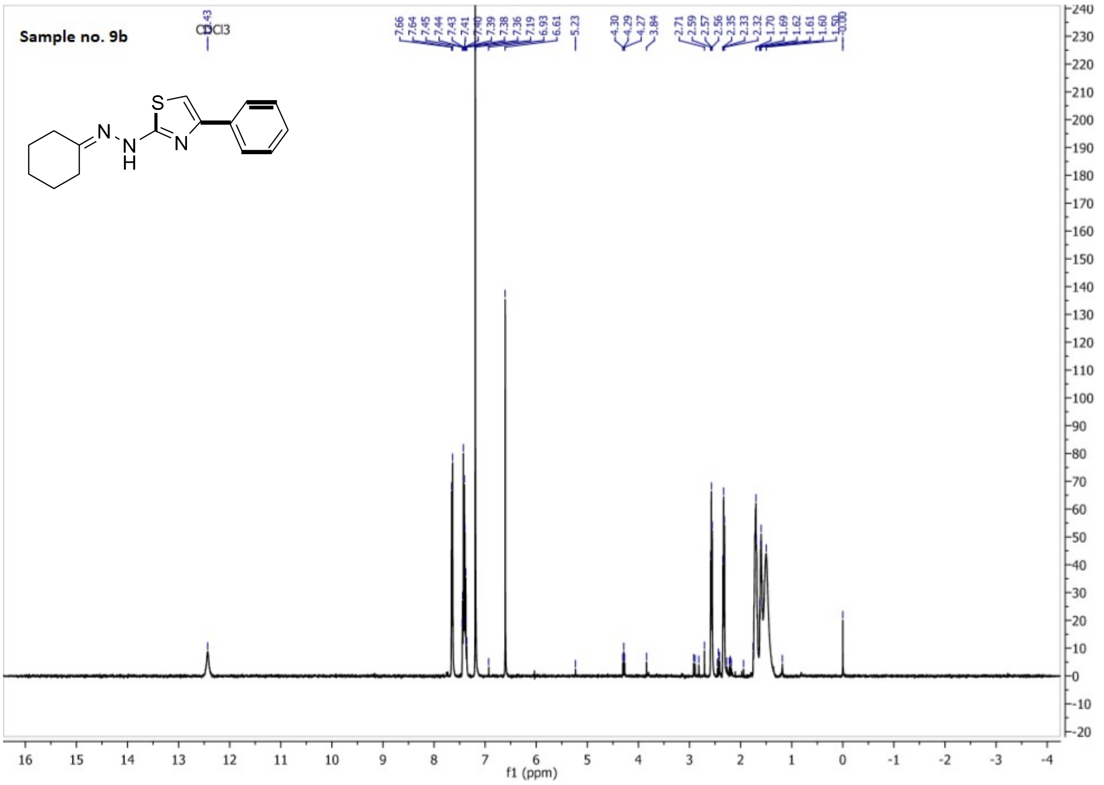

S3 ^{1}H NMR of 9b

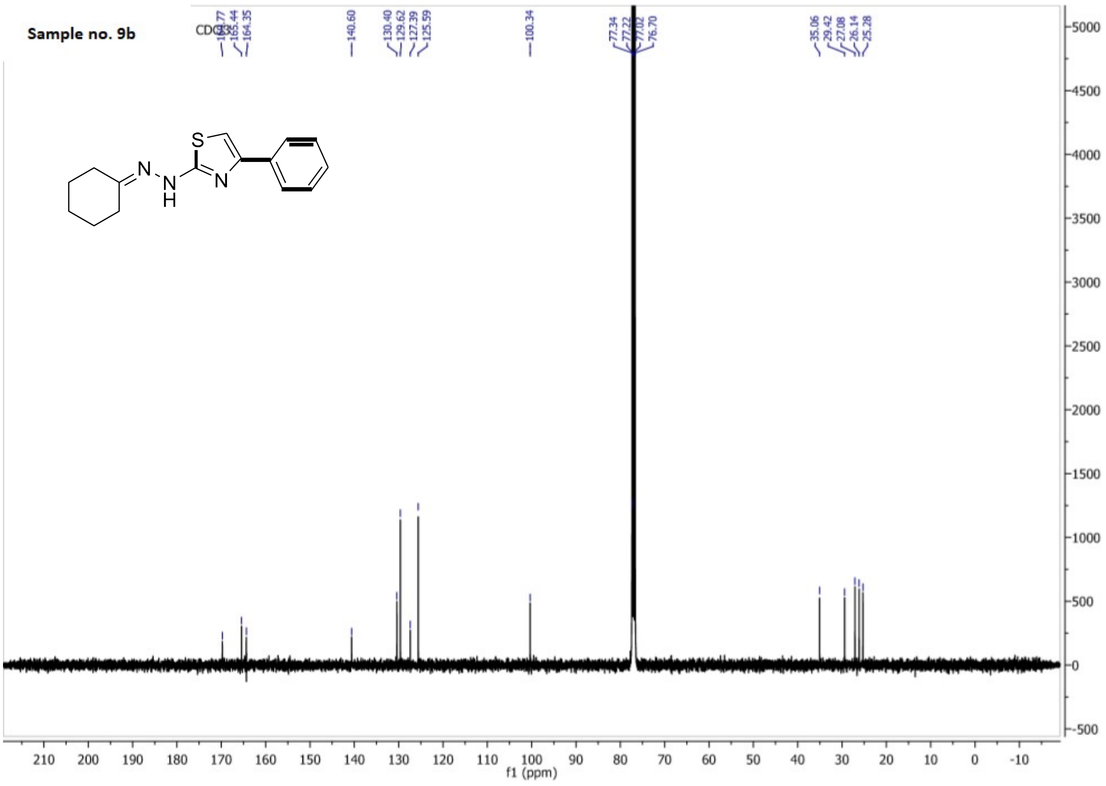

S4 ^{13}C NMR of 9b

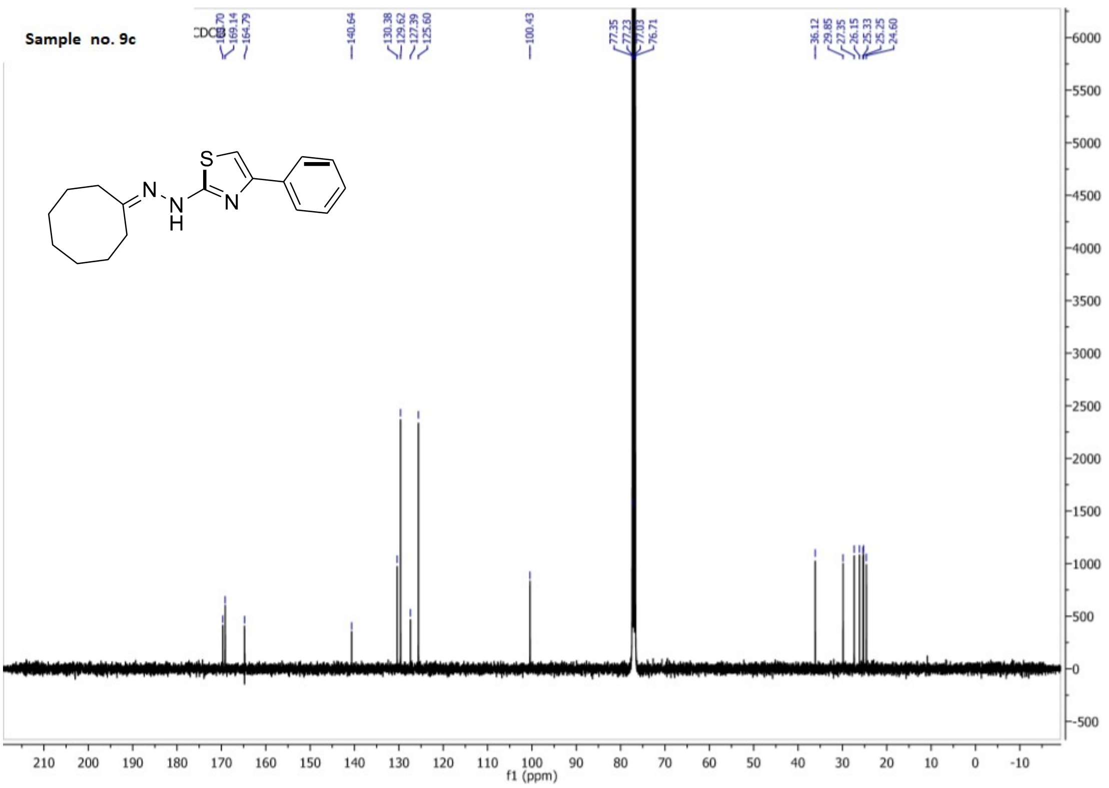

S5 ^{1}H NMR of 9c

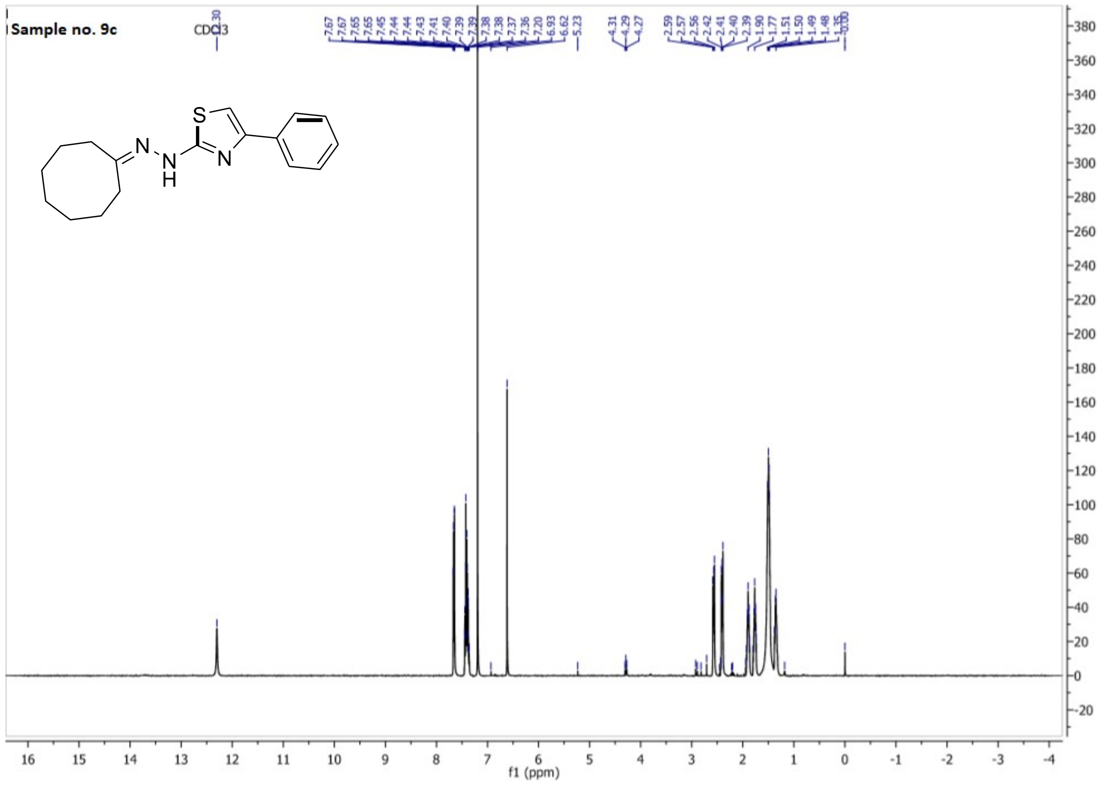

S6 ^{13}C NMR of 9c

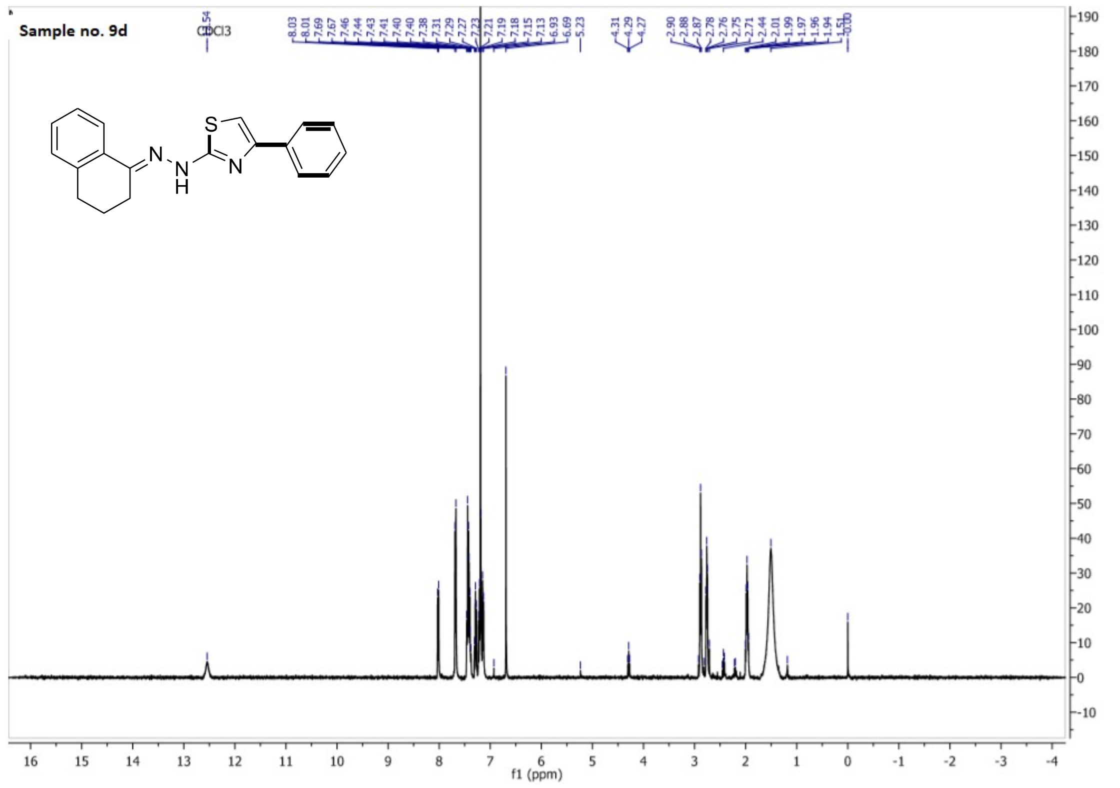

S7 ^{1}H NMR of 9d

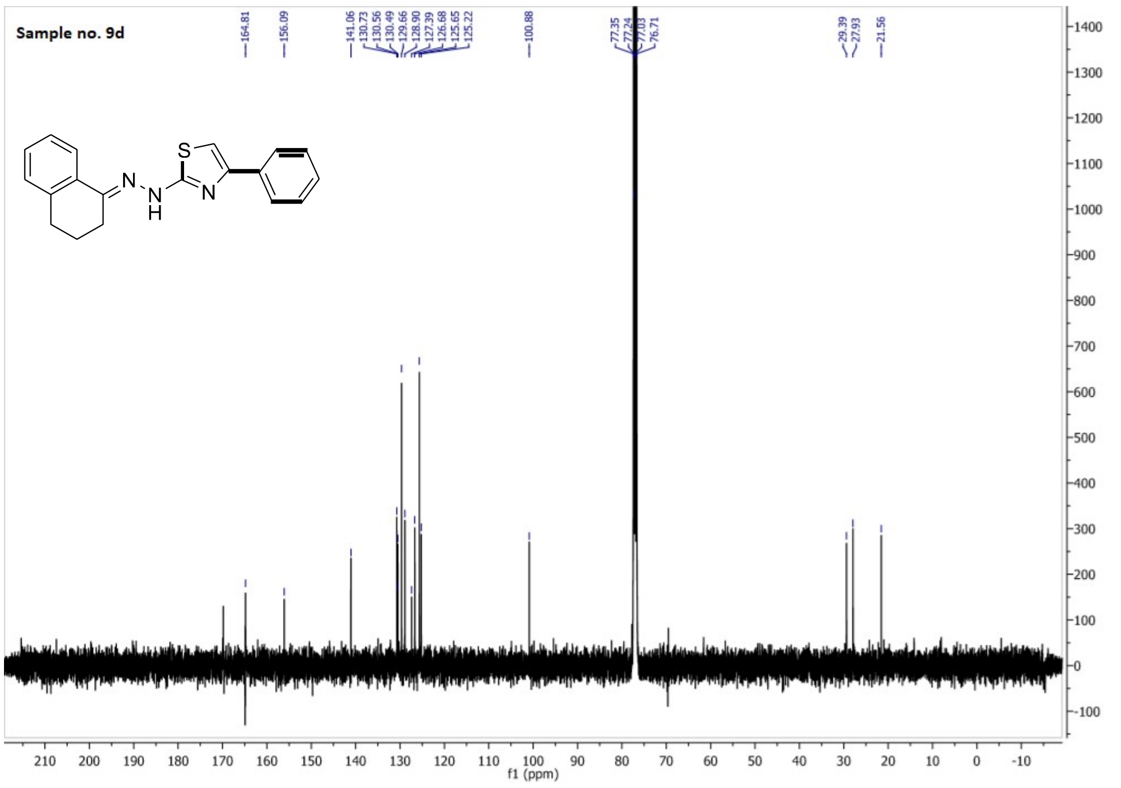

S8 ^{13}C NMR of 9d

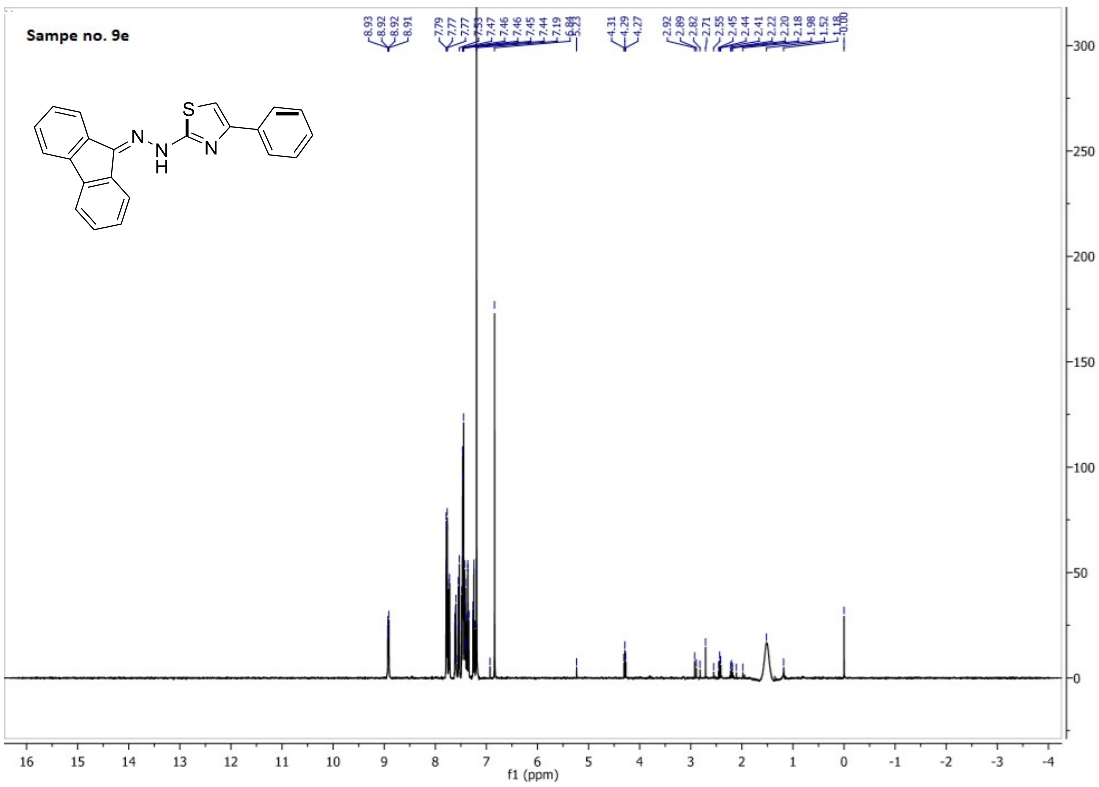

S9 ^{1}H NMR of 9e

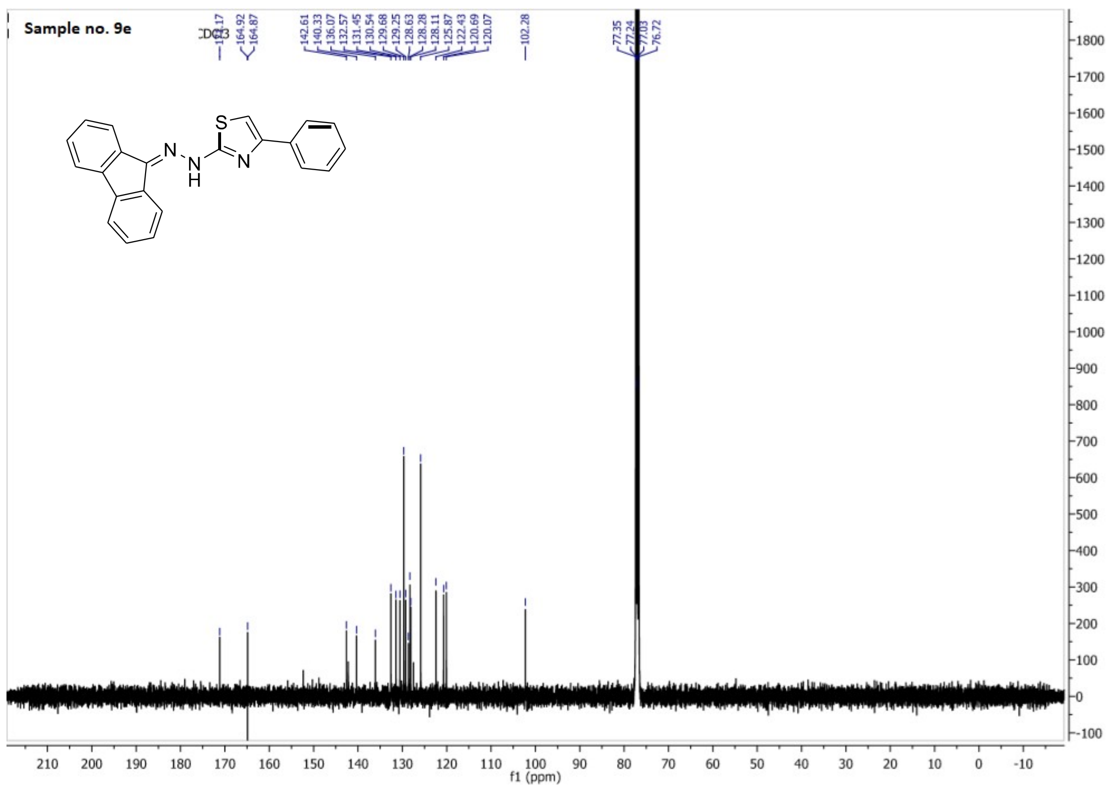

S10 ^{13}C NMR of 9e

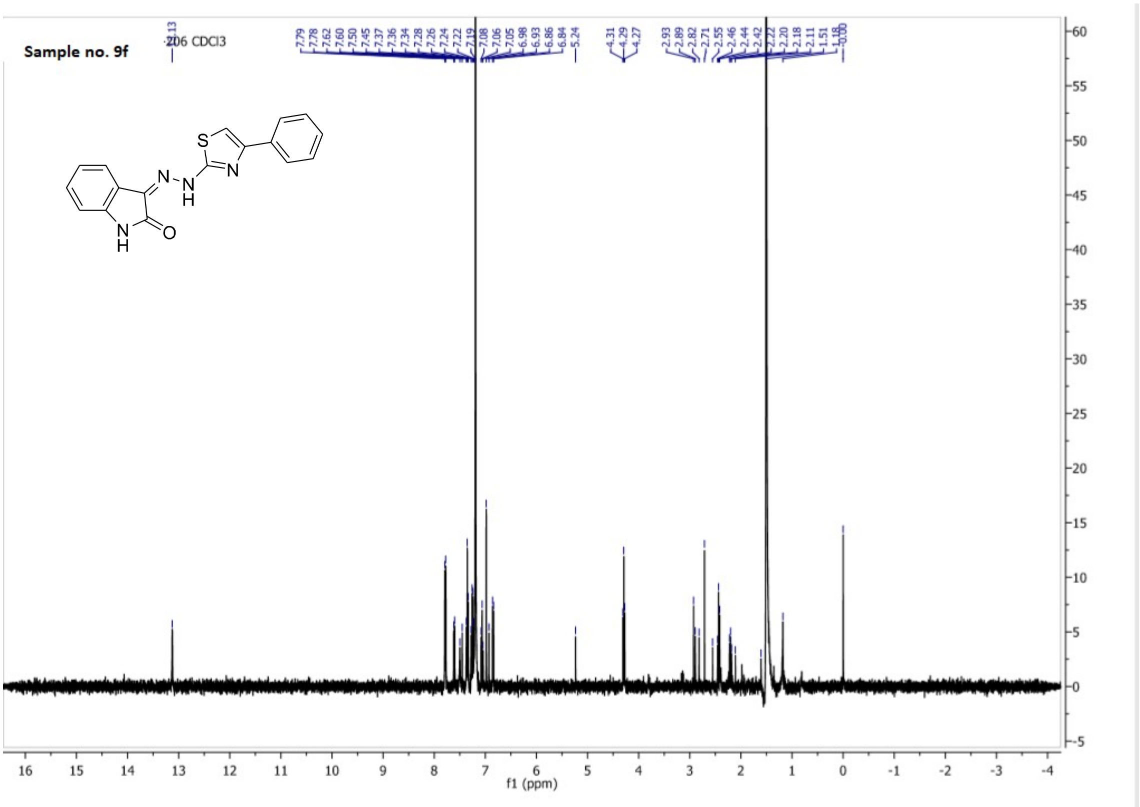

S11 ^{1}H NMR of 9f

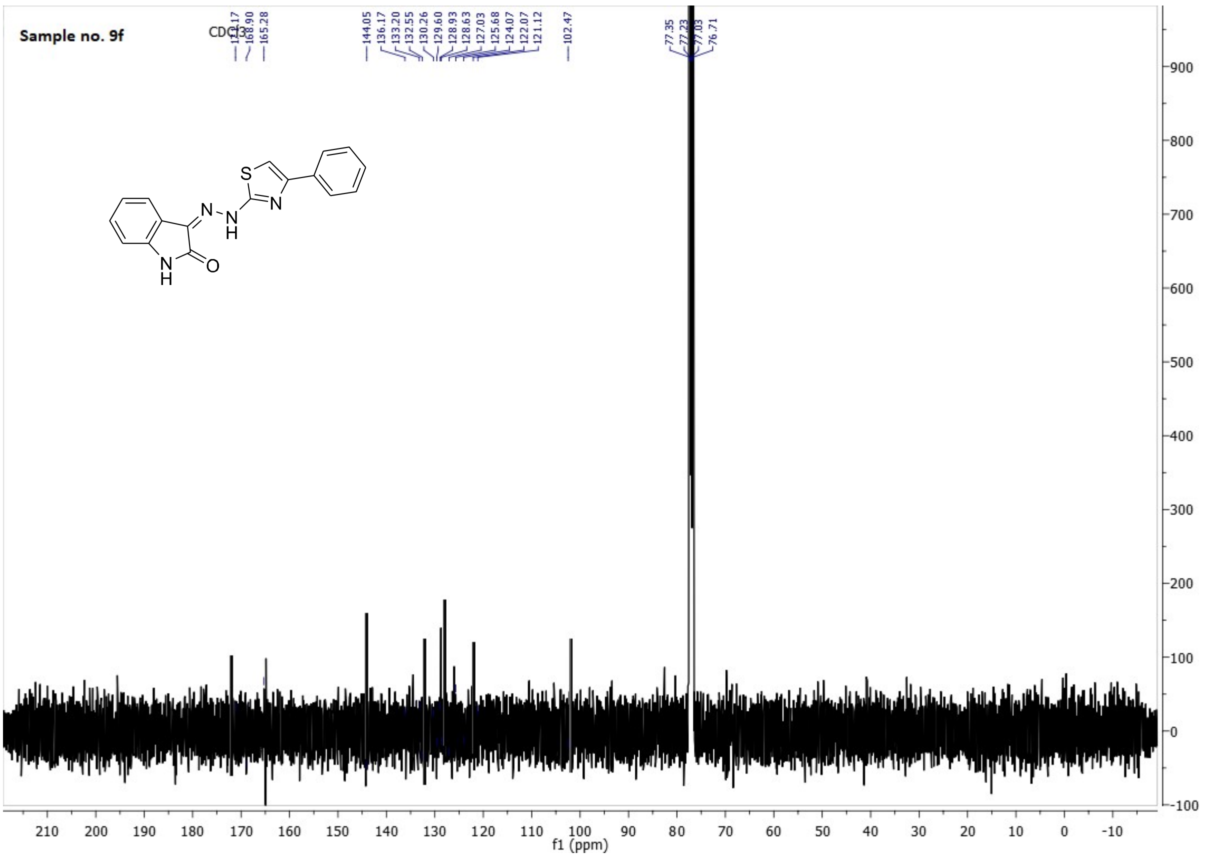

S12 ^{13}C NMR of 9f

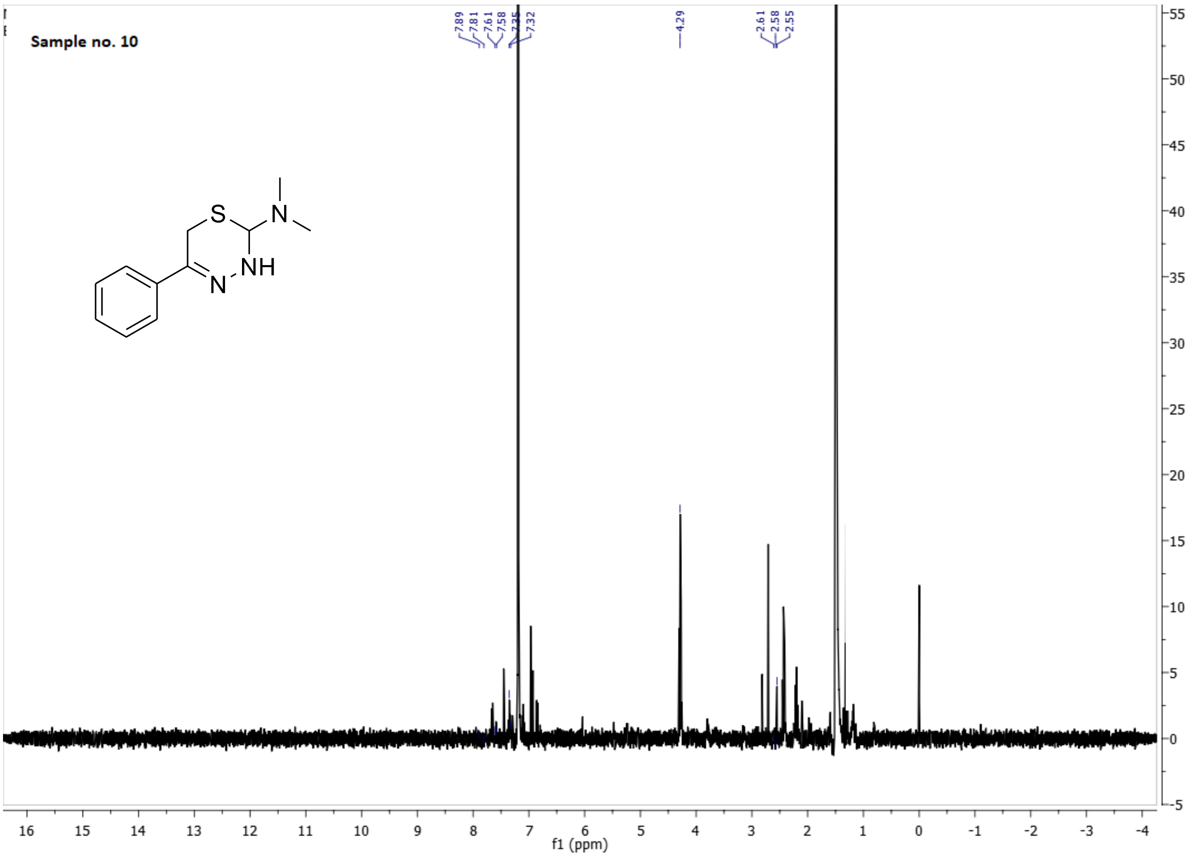

S13 ^{1}H NMR of 10

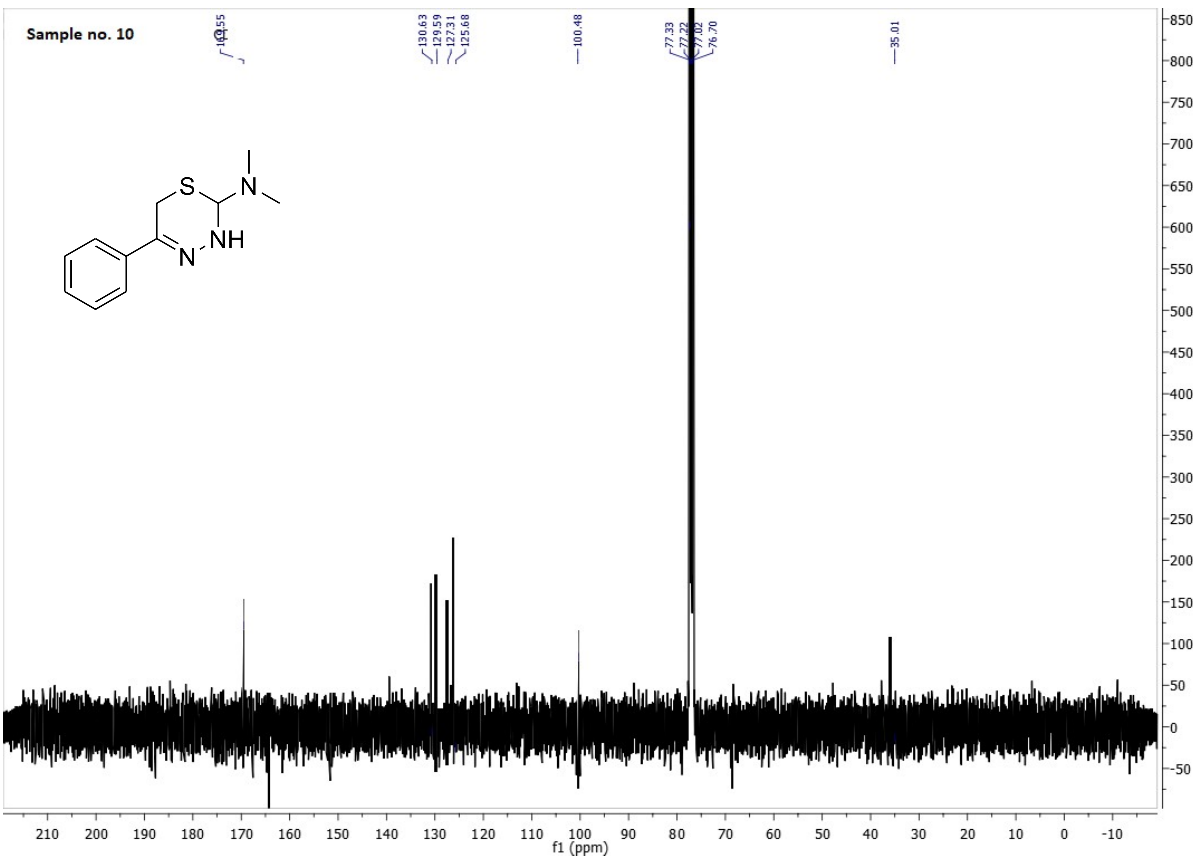

S14 ^{13}C NMR of 10

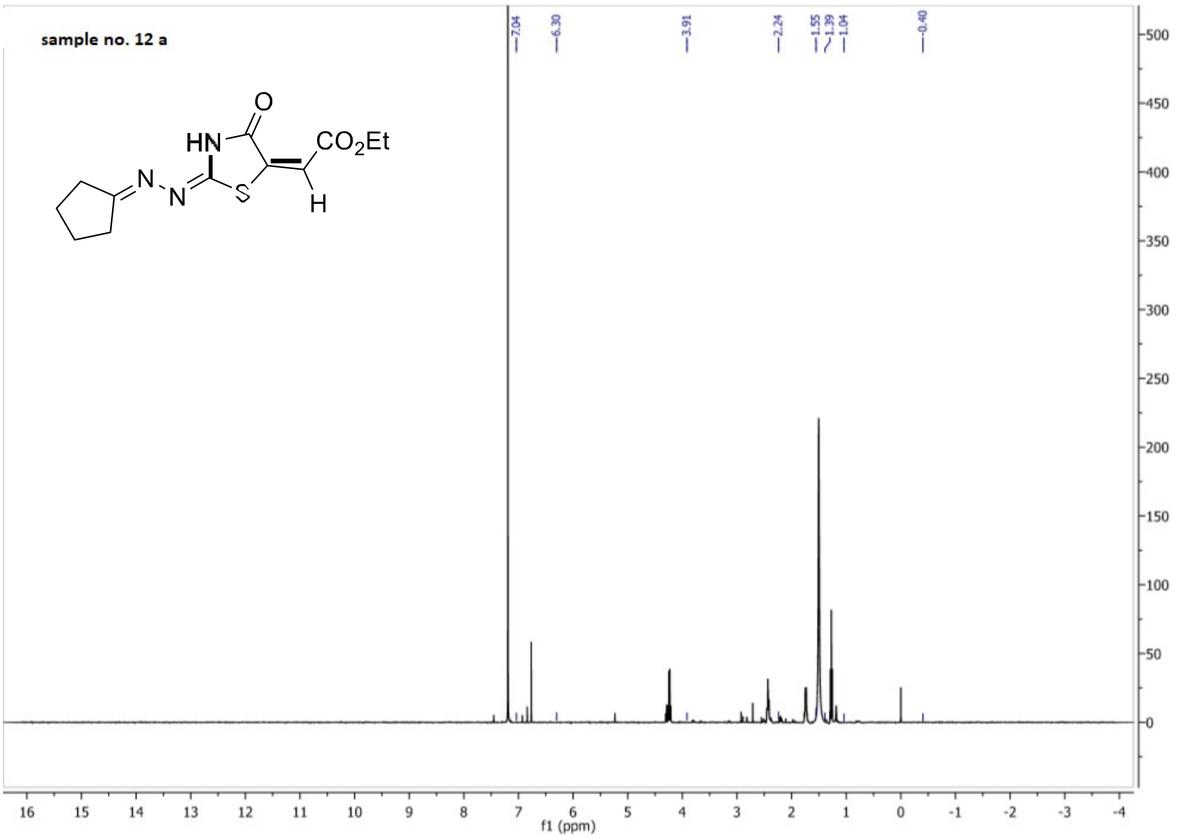

S15 ^{1}H NMR of 12a

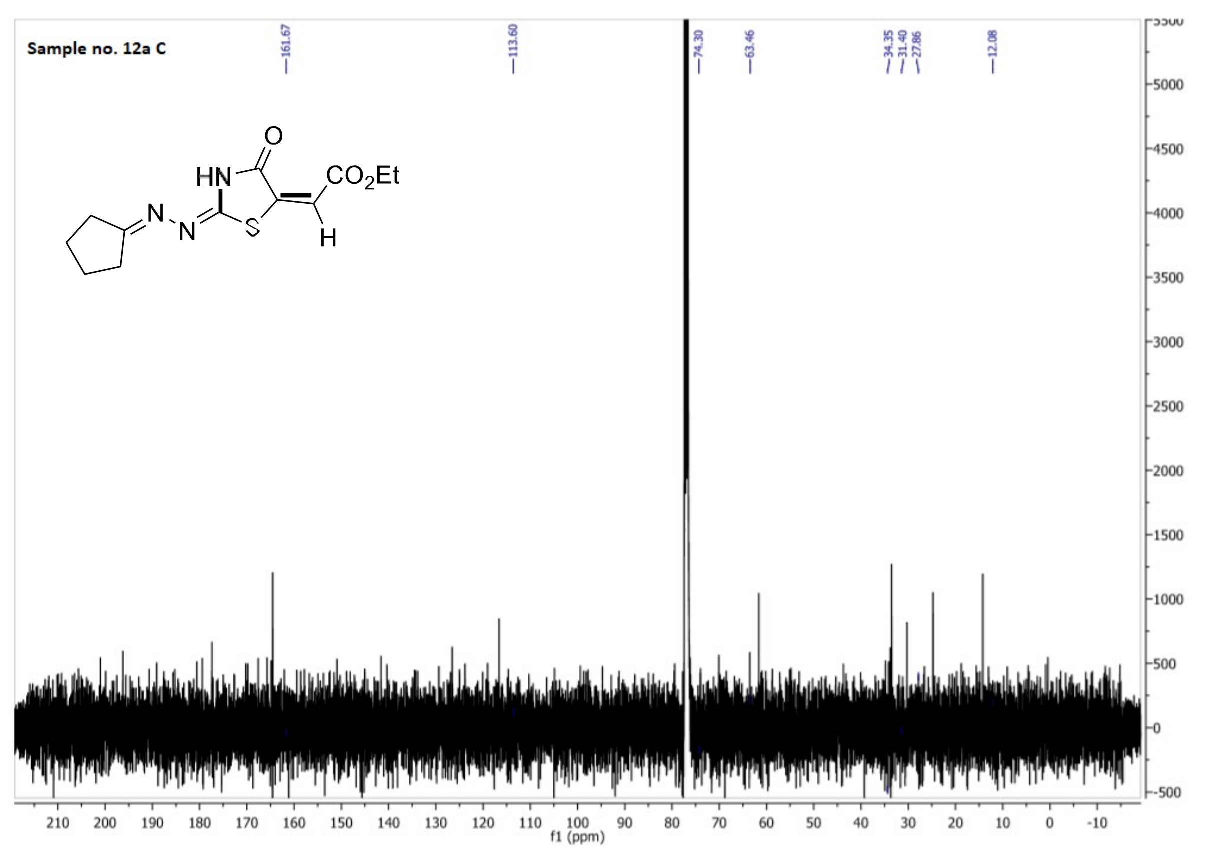

S16 ^{13}C NMR of 12a

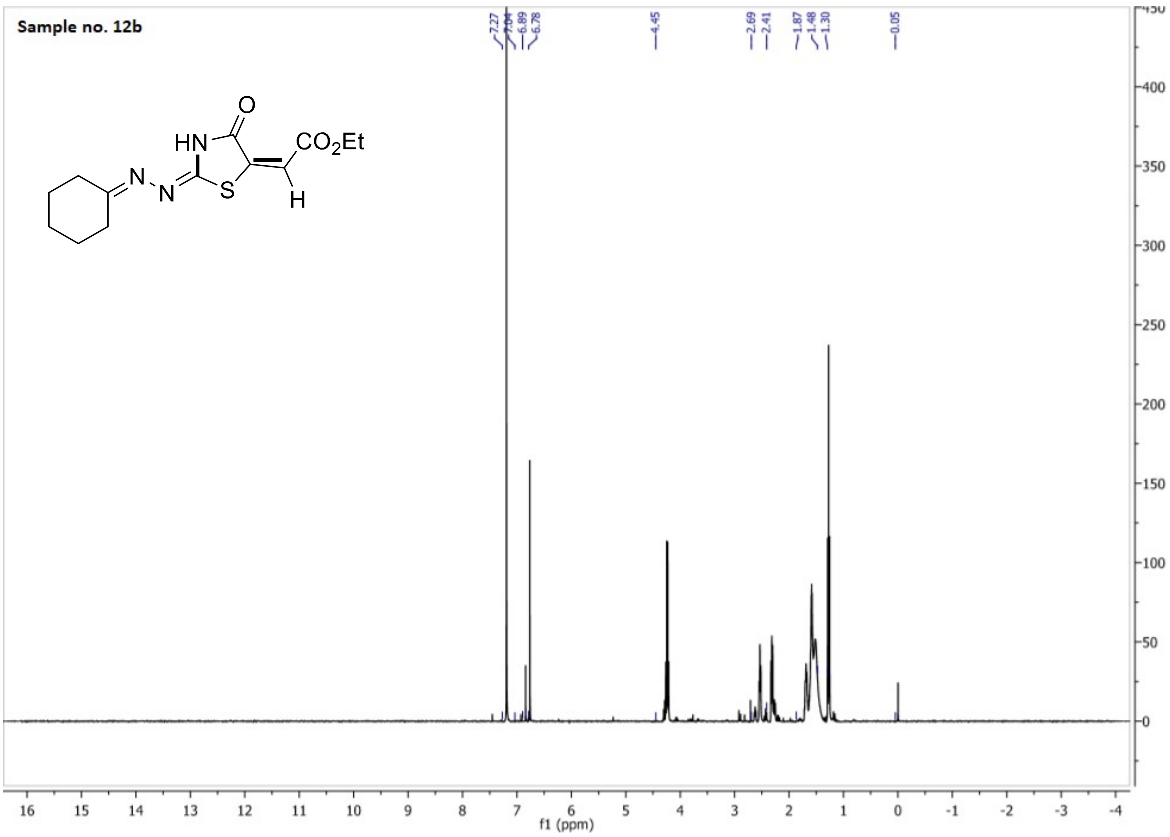

S17 ^{1}H NMR of 12b

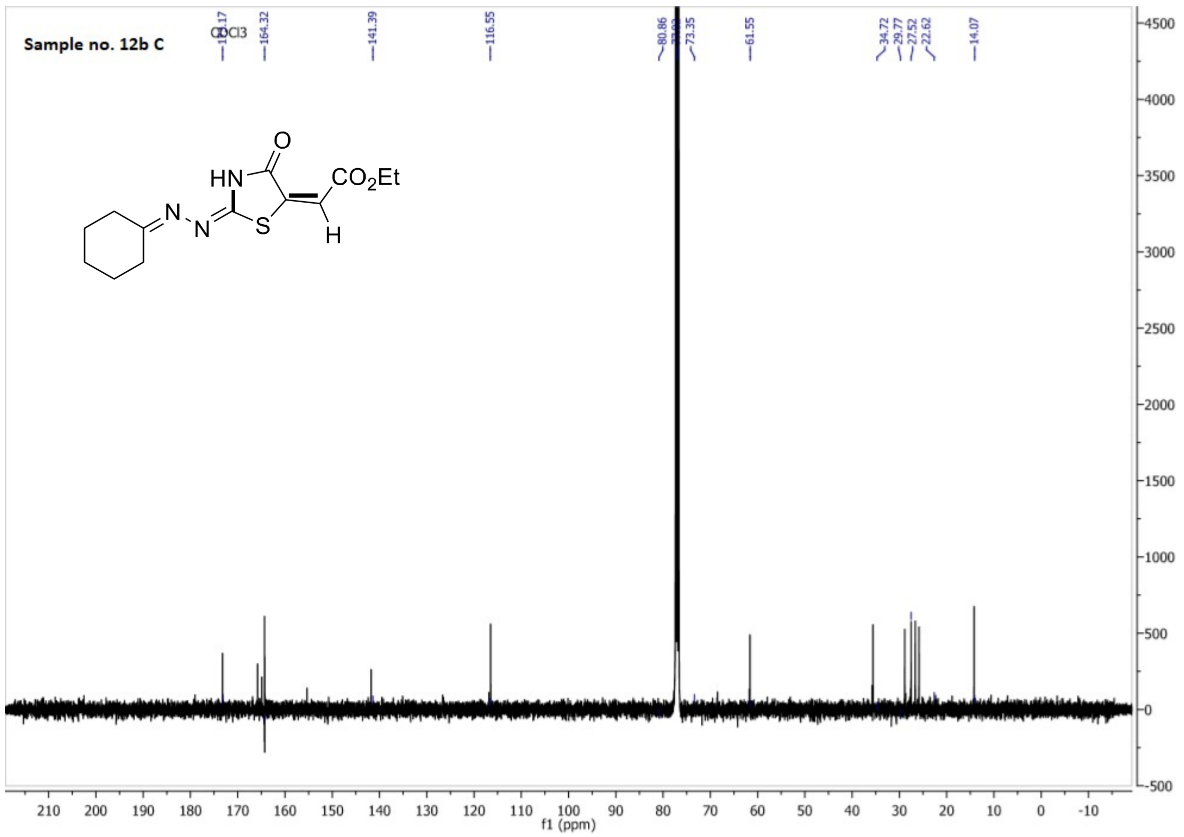

S18 ^{13}C NMR of 12b

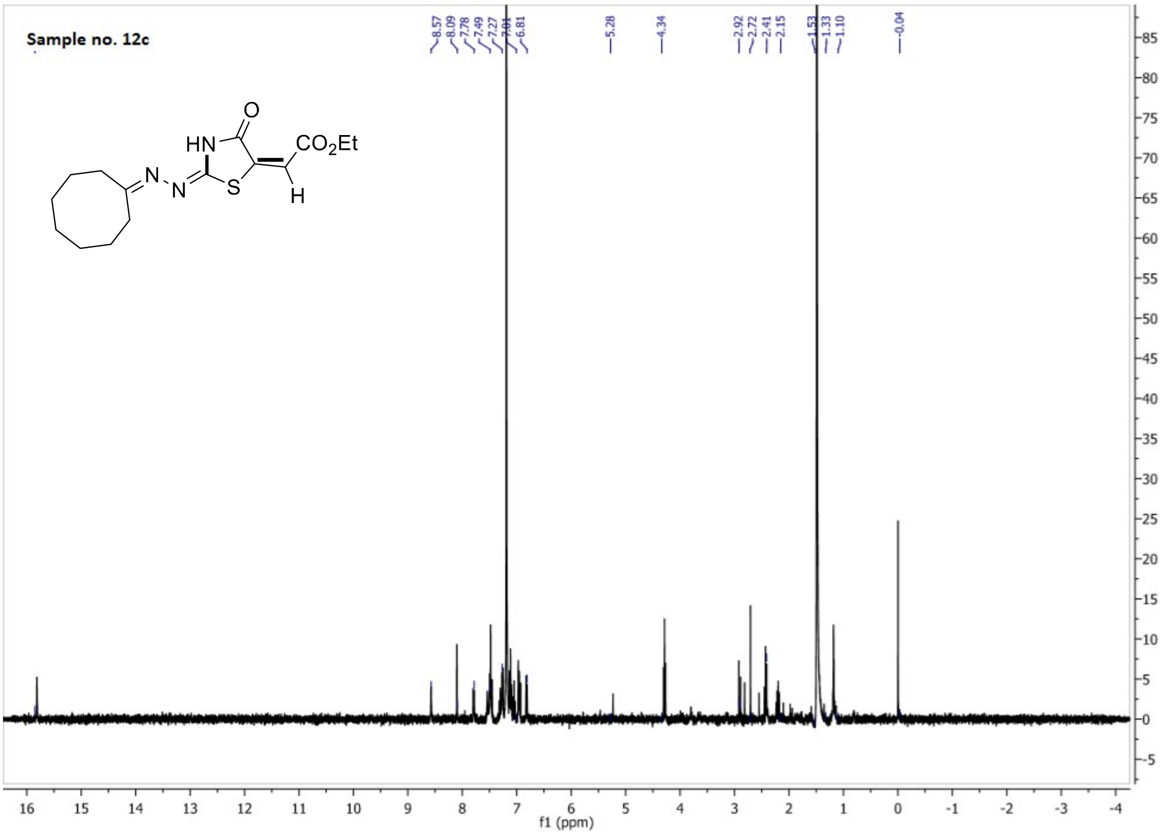

S19 ^{1}H NMR of 12c

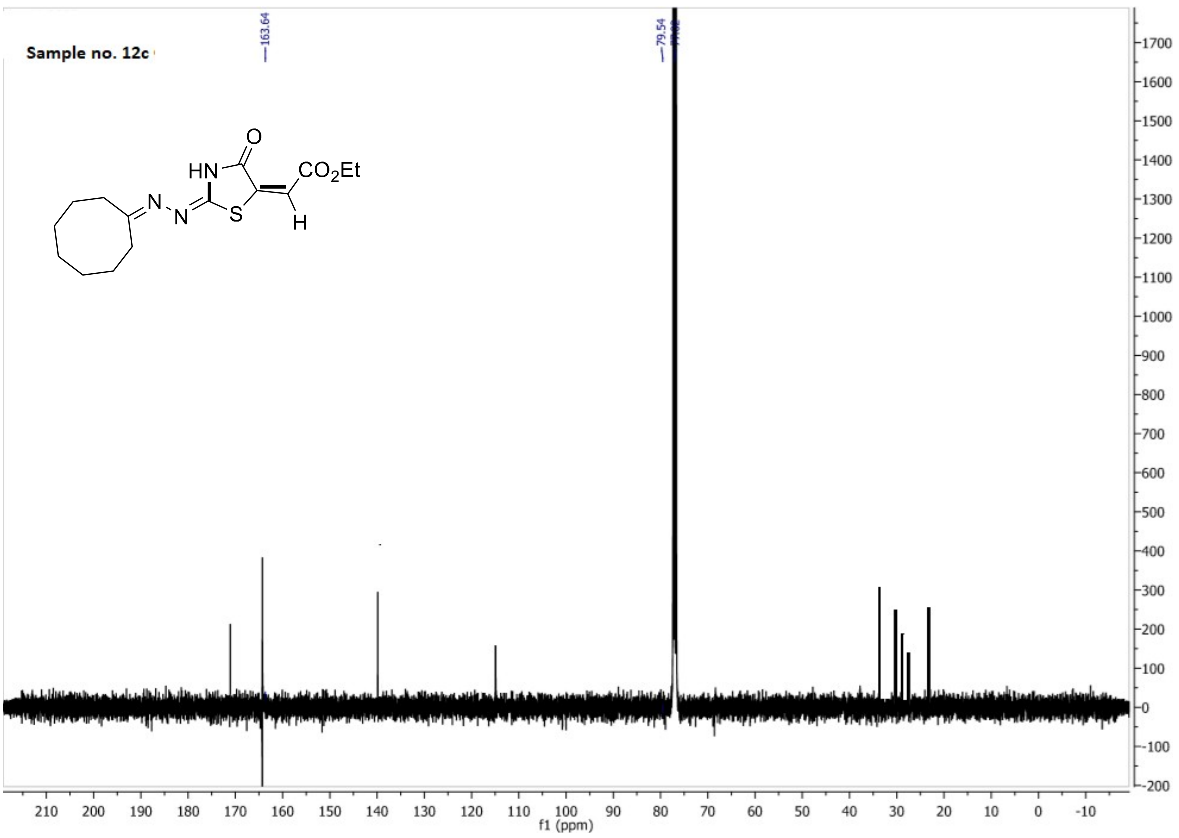

20 ^{13}C NMR of 12c

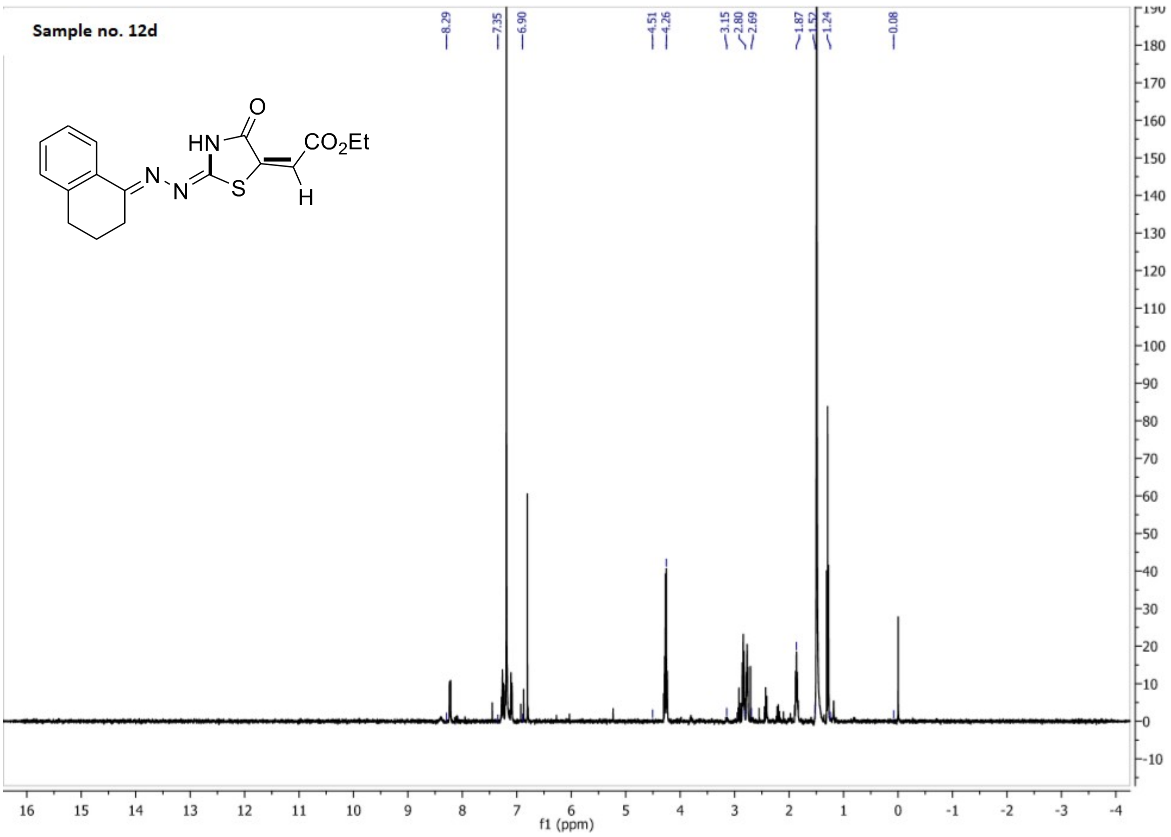

S21 ¹H NMR of 12d

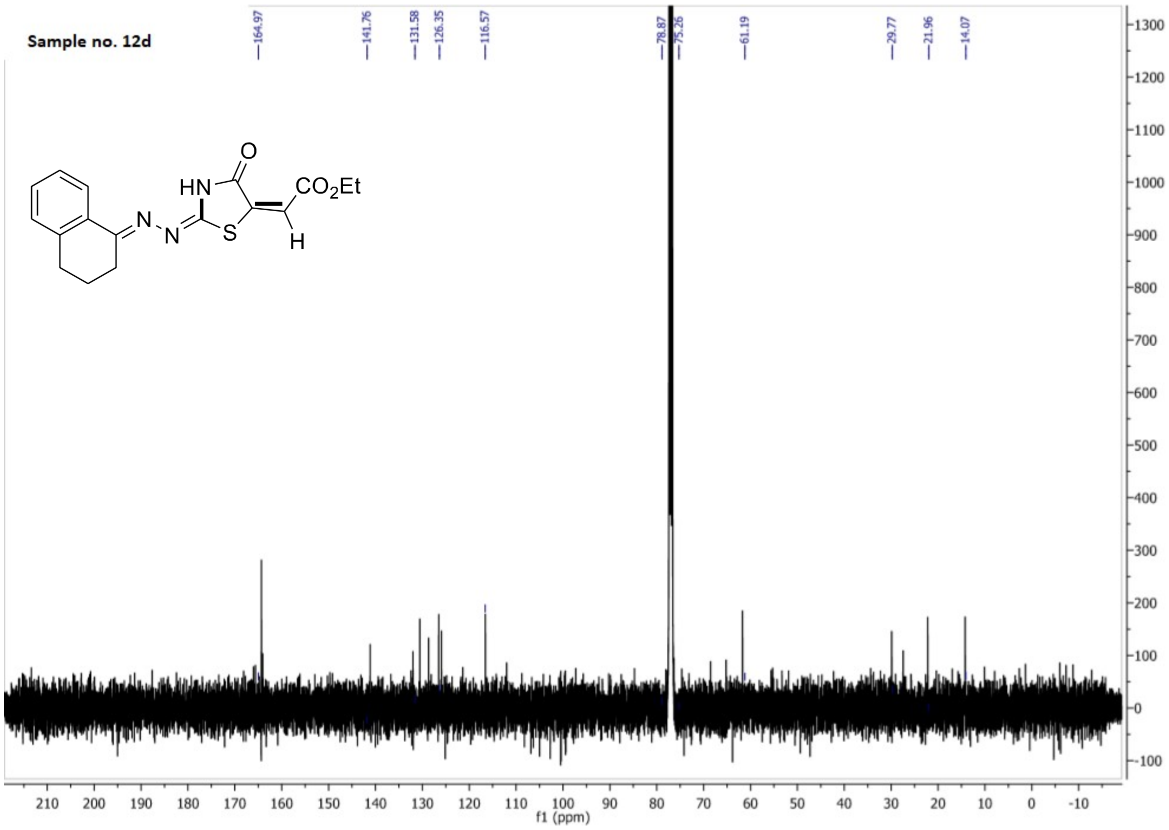

S22 ^{13}C NMR of 12d

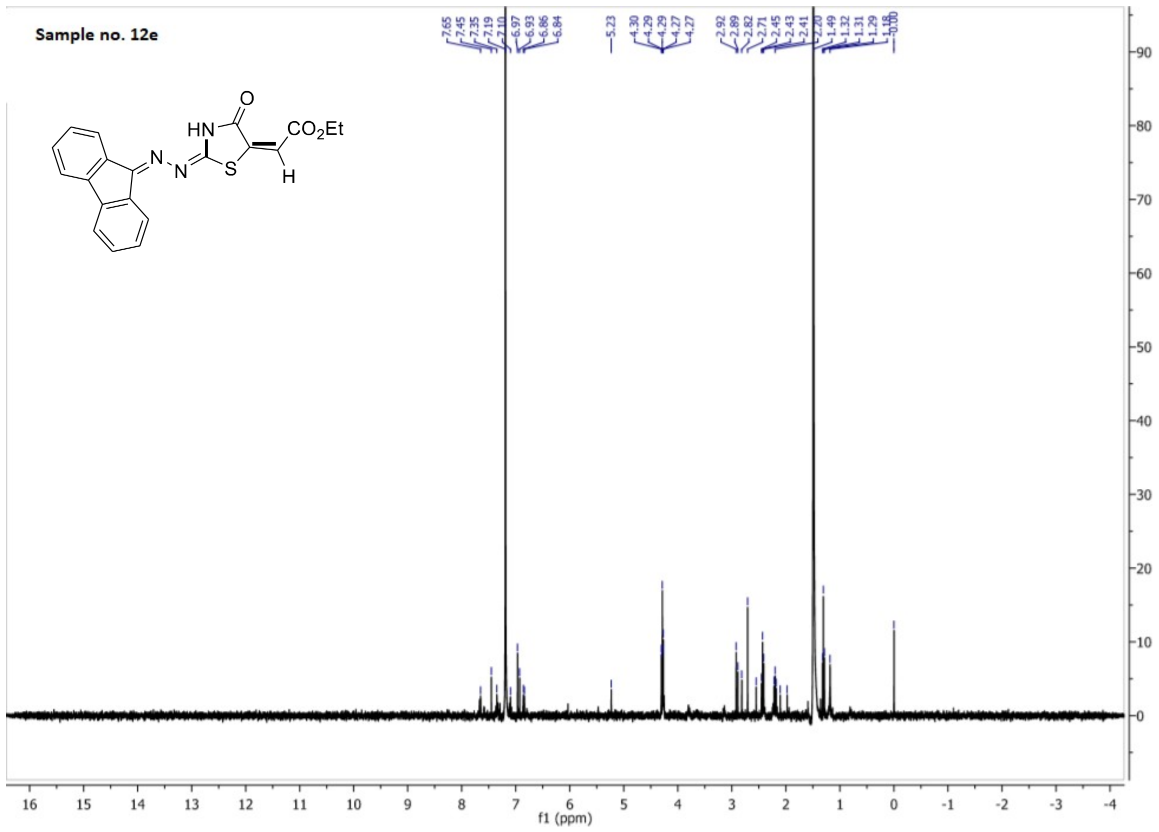

S23 ^{1}H NMR of 12e

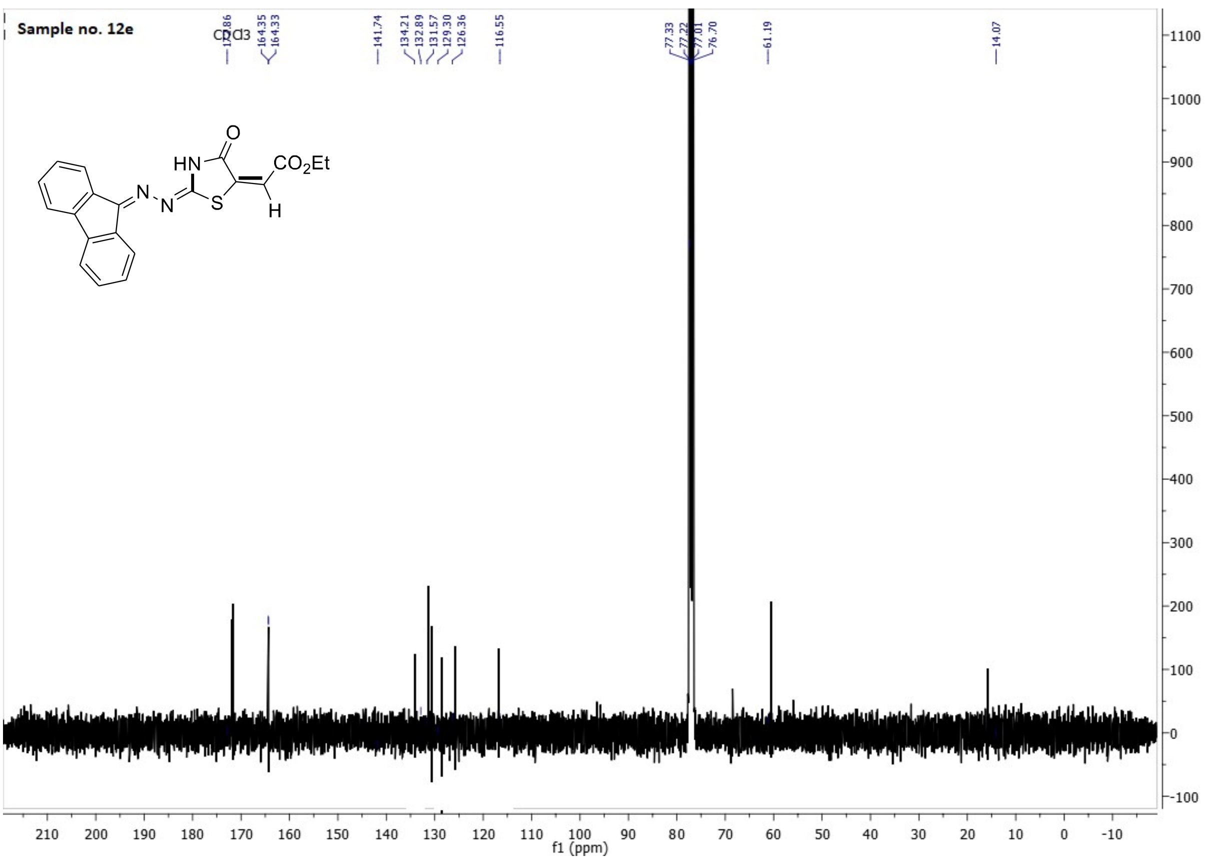

S24 ^{13}C NMR of 12e

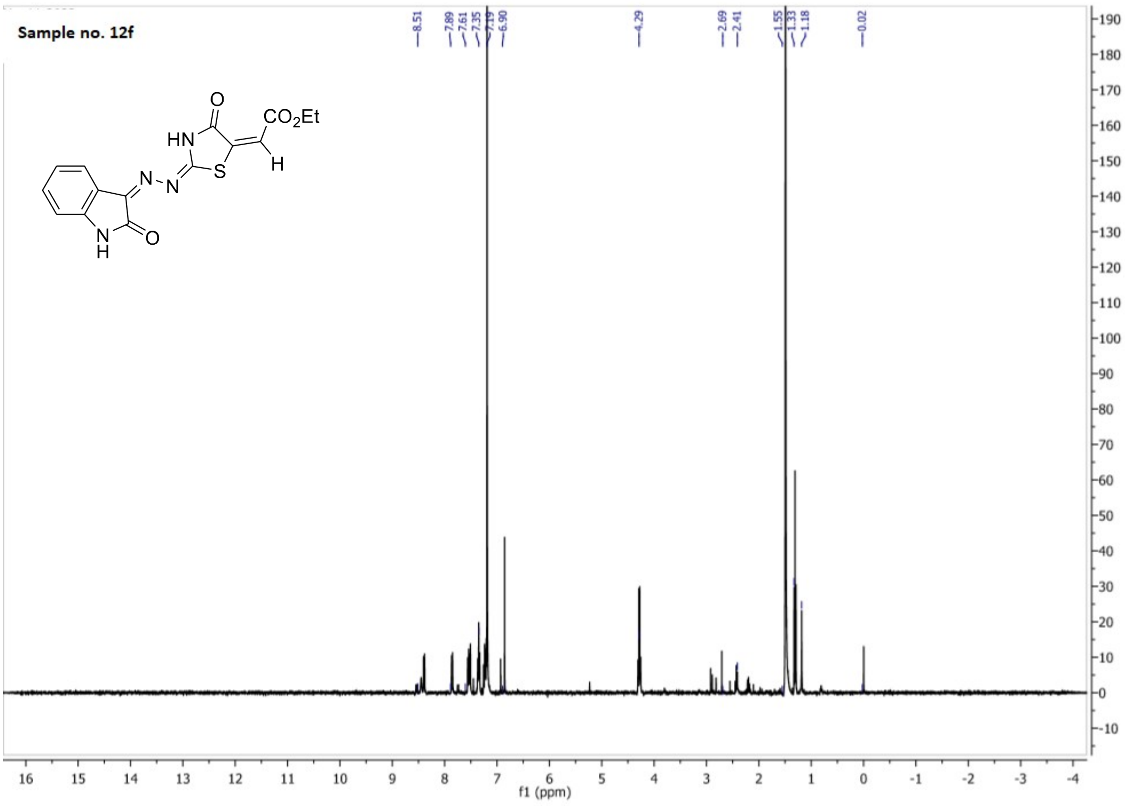

S25 ^{1}H NMR of 12f

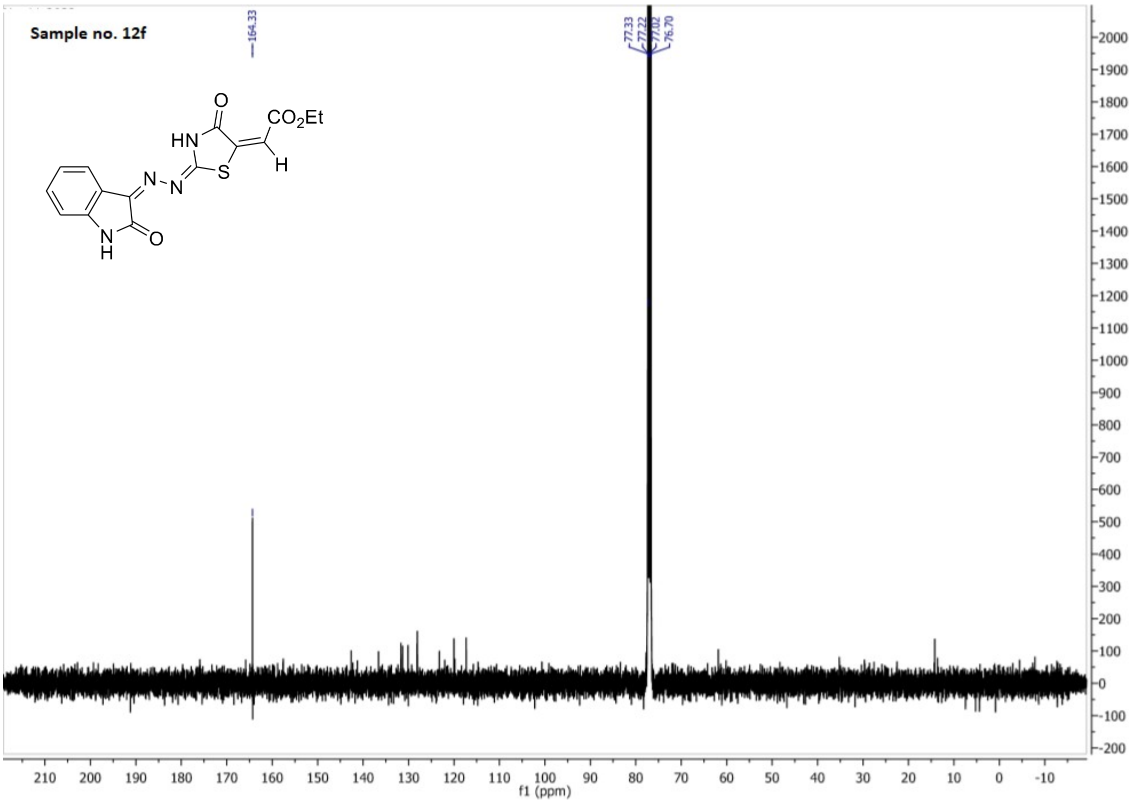

S26 ^{13}C NMR of 12f

4.2. Biological evaluation

4.2.1. Cytotoxic activity using MTT Assay and evaluation of IC_{50}

4.2.1.1. MTT assay

An MTT assay was performed to investigate the effect of the synthesized compounds on mammary epithelial cells (MCF-10A). The cells were propagated in medium consisting of Ham's F-12 medium/ Dulbecco's modified Eagle's medium (DMEM) (1:1) supplemented with 10% fetal calf serum, 2 mM glutamine, insulin (10 μg/mL), hydrocortisone (500 ng/mL), and epidermal growth factor (20 ng/mL). Trypsin ethylenediamine tetra acetic acid (EDTA) was used to passage the cells after every 2-3 days. 96-well flat-bottomed cell culture plates were used to seed the cells at a density of 104 cells mL^{-1}. The medium was aspirated from all the wells of culture plates after 24 h, followed by the addition of synthesized compounds (in 200 μL medium to yield a final concentration of 0.1% (v/v) dimethyl sulfoxide) into individual wells of the plates. Four wells were designated to a single compound. The plates were allowed to incubate at 37 °C for 96 h. Afterwards, the medium was aspirated and 3-[4,5-dimethylthiazol-2-yl]-2,5-diphenyltetrazolium bromide (MTT) (0.4 mg/mL) in medium was added to each well and, subsequently, incubated for 3 h. The medium was aspirated and 150 μL dimethyl sulfoxide (DMSO) was added to each well. The plates were vortexed followed by the measurement of absorbance at 540 nm on a microplate reader. The results were presented as inhibition (%) of proliferation, in contrast to controls comprising 0.1% DMSO.

4.2.1.2. Assay for antiproliferative effect

To explore the antiproliferative potential of compounds, a propidium iodide fluorescence assay was performed using different cell lines, such as Panc-1 (the pancreas cancer cell line), MCF-7 (the breast cancer cell line), HT-29 (the colon cancer cell line), and A-549 (the epithelial cancer cell line). To calculate the total nuclear DNA, a fluorescent dye (propidium iodide, PI) was used, which could attach to the DNA, thus offering a quick and precise technique. PI cannot pass through the cell membrane and its signal intensity can be considered as directly proportional to the quantity of cellular DNA. Cells with cell membranes that were damaged or had changed permeability were counted as dead cells. The assay was performed by seeding the cells of different cell lines at a density of 3000-7500 cells/well (in200µl medium) in culture plates, followed by incubation for 24h at 37 °C in humidified 5%CO2/95% air atmospheric conditions. The medium was removed; the compounds were added to the plates at 10 µM concentrations (in 0.1% DMSO) in triplicate, followed by incubation for 48h. DMSO (0.1%) was used as control. After incubation, the medium was removed, followed by the addition of PI (25 µl, 50µg/mL in water/medium) to each well of the plates. At - 80 °C, the plates were allowed to freeze for 24 h, followed by thawing at 25 °C. A fluorometer (Polar-Star BMG Tech) was used to record the readings at excitation and emission wavelengths of 530 and 620 nm for each well. The percentage cytotoxicity of compounds was calculated using the following formula:

$$\% \text{ Cytotoxicity} = \frac{A_C - A_{TC}}{A_C} \times 100$$

where ATC = absorbance of treated cells and AC = absorbance of control. Erlotinib was used as the positive control in the assay.

4.2.2. EGFR inhibitory assay

Baculoviral expression vectors, including pBlueBacHis2B and pFASTBacHTc, were used separately to clone 1.6 kb cDNA coding for EGFR cytoplasmic domain (EGFR-CD, amino acids 645–1186). 5′ upstream to the EGFR sequence comprised a sequence that encoded (His)6. Sf-9 cells were infected for 72h for protein expression. The pellets of Sf-9 cells were solubilized in a buffer containing sodium vanadate (100 μM), aprotinin (10 μg/mL), triton (1%), HEPES buffer(50mM), ammonium molybdate (10 μM), benzamidine HCl (16 μg/mL), NaCl (10 mM),leupeptin (10 μg/mL), and pepstatin (10 μg/mL) at 0 °C for 20 min at pH 7.4, followed by centrifugation for 20 min. To eliminate the nonspecifically bound material, an Ni-NTA super flow packed column was used to pass through and wash the crude extract supernatant, first with 10mM and, then, with 100 mM imidazole. Histidine-linked proteins were first eluted with 250 and, then, with 500 mM imidazole, subsequent to dialysis against NaCl (50 mM), HEPES (20 mM), glycerol (10%) and 1 μg/mL each of aprotinin, leupeptin and pepstatin for 120 min. The purification was performed either at 4 °C or on ice. To record the autophosphorylation level, an EGFR kinase assay was carried out on the basis of DELFIA/time-resolved fluorometry. The compounds were first dissolved in DMSO absolute, subsequent to dilution to appropriate concentration using HEPES (25 mM) at pH 7.4. Each compound (10 μL) was incubated with recombinant enzyme (10 μL, 5 ng for EGFR, 1:80 dilution in 100 mM HEPES) for 10 min at 25 ºC, subsequent to the addition of 5X buffer (10 μL, containing 2 mM MnCl2, 100 μM Na3VO4, 20 mM HEPES, and 1 mM DTT) and ATP-MgCl2 (20 μL, containing 0.1 mM ATP and 50 mM MgCl2) and incubation for 1h. The negative and positive controls were included in each plate by the incubation of enzyme, either with or without ATP-MgCl2. The liquid was removed after incubation and the plates were washed thrice using wash buffer. Europium-tagged antiphosphotyrosine antibody (75 μL, 400 ng) was

added to each well, followed by incubation of 1h and, then, by the washing of the plates using buffer. The enhancement solution was added to each well and the signal was recorded at excitation and emission wavelengths of 340 at 615 nm, respectively. The autophosphorylation percentage inhibition by compounds was calculated using the following equation:

$$100\% - [(negative\ control)/(positive\ control) - (negative\ control)]$$

Using the curves of percentage inhibition of eight concentrations of each compound, IC50 was calculated. Majority of signals detected by antiphosphotyrosine antibody were from EGFR, because the enzyme preparation contained low impurities.

4.2.3. BRAF^{V600E} inhibitory assay

A V^{600E} mutant BRAF kinase assay was performed to investigate the activity of tested compounds against BRAF. Mouse full-length GST-tagged BRAF^{V600E} (7.5 ng, Invitrogen, PV3849) was pre- incubated with drug (1 µL) and assay dilution buffer (4 µL) for 60 min at 25 ºC. In assay dilution buffer, a solution (5 µL) containing $MgCl_2$ (30 mM), ATP (200 µM), recombinant human full length (200 ng) and *N*-terminal His-tagged MEK1 (Invitrogen) was added to start the assay, subsequent to incubation for 25 min at 25 ºC. The assay was stopped using 5X protein denaturing buffer (LDS) solution (5 µL). To further denature the protein, heat (70° C) was applied for 5 min. 4-12% precast NuPage gel plates (Invitrogen) were used to carry out electrophoresis (at 200 V). Ten µL of each reaction was loaded into the precast plates and electrophoresis was allowed to proceed. After completion of electrophoresis, the front part of the precast gel plate (holding hot ATP) was cut, and then cast-off. The dried gel was developed using a phosphor screen. A reaction without active enzyme was used as the negative control, while that containing no inhibitor served as the positive control. To study the effect of compounds on cell-based pERK1/2 activity in

cancer cells, commercially available ELISA kits (Invitrogen) were used, according to manufacturer's instructions.

4.3. Statistical analysis

The computerized Prism 5 program was used to statistically analyze data, using a one-way ANOVA test, followed by Tukey's as post-ANOVA for multiple comparison at $p \leq .05$. Data were presented as mean ± SEM.
